# Microbial food web components, bulk metabolism, and single-cell physiology of piconeuston in surface microlayers of high-altitude lakes

**DOI:** 10.3389/fmicb.2015.00361

**Published:** 2015-05-05

**Authors:** Hugo Sarmento, Emilio O. Casamayor, Jean-Christophe Auguet, Maria Vila-Costa, Marisol Felip, Lluís Camarero, Josep M. Gasol

**Affiliations:** ^1^Institut de Ciències del Mar, ICM-Consejo Superior de Investigaciones CientíficasBarcelona, Spain; ^2^Laboratory of Microbial Processes and Biodiversity, Department of Hydrobiology, Federal University of São CarlosSão Carlos, Brazil; ^3^Integrative Freshwater Ecology Group, Limnological Observatory of the Pyrenees, Centre d'Estudis Avançats de Blanes, CEAB-Consejo Superior de Investigaciones CientíficasBlanes, Spain; ^4^Department of Environmental Chemistry, IDAEA-CSICBarcelona, Spain; ^5^Centre de Recerca d'Alta Muntanya, Universitat de BarcelonaLleida, Spain

**Keywords:** picoplankton, leucine incorporation, bacterioplankton, flagellates, flow cytometry, NADS, CTC, ultraviolet radiation

## Abstract

Sharp boundaries in the physical environment are usually associated with abrupt shifts in organism abundance, activity, and diversity. Aquatic surface microlayers (SML) form a steep gradient between two contrasted environments, the atmosphere and surface waters, where they regulate the gas exchange between both environments. They usually harbor an abundant and active microbial life: the neuston. Few ecosystems are subjected to such a high UVR regime as high altitude lakes during summer. Here, we measured bulk estimates of heterotrophic activity, community structure and single-cell physiological properties by flow cytometry in 19 high-altitude remote Pyrenean lakes and compared the biological processes in the SML with those in the underlying surface waters. Phototrophic picoplankton (PPP) populations, were generally present in high abundances and in those lakes containing PPP populations with phycoerythrin (PE), total PPP abundance was higher at the SML. Heterotrophic nanoflagellates (HNF) were also more abundant in the SML. Bacteria in the SML had lower leucine incorporation rates, lower percentages of “live” cells, and higher numbers of highly-respiring cells, likely resulting in a lower growth efficiency. No simple and direct linear relationships could be found between microbial abundances or activities and environmental variables, but factor analysis revealed that, despite their physical proximity, microbial life in SML and underlying waters was governed by different and independent processes. Overall, we demonstrate that piconeuston in high altitude lakes has specific features different from those of the picoplankton, and that they are highly affected by potential stressful environmental factors, such as high UVR radiation.

## Introduction

The upper micrometers of the water column of aquatic ecosystems form a substantially different environment than that of the underlying surface water, despite its close proximity as reviewed by Cunliffe et al. (2011). The group of organisms living at the water-air interface, the surface microlayer (SML), was named neuston (Naumann, 1917), and the smallest organisms (<2 μm) living there can be defined as piconeuston. The SML is the boundary between two totally different environments and the site where fundamental biological and chemical processes, such as gas exchanges between atmosphere and water occur (e.g., Conrad and Seiler, 1988). The SML is known to concentrate substances, which may enhance or inhibit microbial activities. Previous studies showed that the marine SML was enriched with amino acids and bacteria and, in most instances, virus-like particles (Kuznetsova et al., 2004), and that heterotrophic piconeuston had enhanced extracellular enzymatic peptide hydrolysis rates (Kuznetsova and Lee, 2001), compared to subsurface waters.

However, and at least in marine/estuarine environments, early reports indicated that heterotrophic piconeuston has lower metabolic activity than surface picoplankton, (Dietz et al., 1976). This difference in metabolism was explained as a community response to different environmental stress factors occurring preferentially in the SML, such as high incident solar UVR, and more variable water temperature and salinity, as well as potentially higher concentrations of toxic substances (Maki, 1993). In aquatic environments naturally subjected to high UVR doses, such as high altitude lakes in summer, a difference in activity should therefore be expected.

The differences in metabolism, however, might also be attributed to differences in community compositions between the SML and the surface. Auguet and Casamayor (2008), using catalyzed reporter deposition fluorescence *in situ* hybridization (CARD-FISH) and 16S rRNA gene sequence analysis, reported a higher abundance of archaeal communities in the SML of Pyrenean oligotrophic high-mountain lakes as compared to surface communities. These Archaea populations were composed mainly of Crenarchaeota, whereas surface populations were mainly comprised of Euryarchaeota. Similarly, Vila-Costa et al. (2014) found distinctive populations of both archaea and bacteria inhabiting SML and surface waters of the same Pyrenean lakes using 454 pyrosequencing, and the differences were exacerbated under atmospheric loadings that stimulated microbial activities. A less clear pattern was observed in a set of six Alpine lakes located across an altitude gradient (Hörtnagl et al., 2010), where Betaproteobacteria (enumerated by CARD-FISH) dominated in both SML and underlying water, and the differences observed among lakes were attributed to lake-specific intrinsic factors.

Living in the SML is rather challenging, mainly due to the harsh prevailing conditions resulting from summer extreme UVR levels (Sommaruga, 2001). Previous reports indicating that UVR negatively affects bacterial activity (i.e., Ruiz-González et al., 2013), HNF growth, and bacterial consumption rates (Sommaruga et al., 1999) suggest that microorganisms living in the neuston should experience heavy environmental stress. Independently of the peculiarity of SML's prokaryotic taxonomic composition described in the studies cited above, there is little information available on the *in situ* microbial food web structure (i.e., both abundance and composition of heterotrophic prokaryotes, phototrophic picoplankton [PPP], and heterotrophic nanoflagellates [HNF]) and of bacterial single-cell activity and physiology, which could illustrate the ecological processes shaping life in the SML.

The aim of this study was to study microbial community structure, metabolism, and physiology of piconeuston of SML compared to underlying water in high mountain lakes. Our working hypothesis is that microbial communities living in the SML of high altitude lakes are subjected to environmental harshness that affects their composition, community structure, activity, and physiology in a different way than that of surface waters communities. In order to achieve this goal, we carried out a comprehensive flow-cytometry measurement of (i) microbial community structure, (ii) prokaryotic bulk and single-cell activity, and (iii) physiological status in 19 remote high altitude lakes sampled under summer high solar radiation conditions, in order to determine the variability of these parameters in the SML as compared to surface waters. To the best of our knowledge, most of the variables studied, such as detailed microbial community structure by flow cytometry and bacterial single-cell activity, had never before been measured in the SML.

## Materials and methods

### Sampling sites and limnological parameters

A set of 19 high mountain lakes from the Central Pyrenees were sampled from 17th to 24th, June 2008 at 3 depths: in the first ~400 μm of the water column, here defined as the SML; at 0.5 m depth—which we label as “surface”; and at the depth equivalent to 1.5-fold Secchi disk value, usually corresponding to the depth of the summer deep chlorophyll maximum (DCM) (Catalan et al., 2006), which ranged from 2 to 30 m depth, depending on the lake. In this report the DCM values of Chlorophyll-*a* (Chla) were only used to characterize the lakes according to their nutrient and trophic status. In these clear water mountain lakes Chla at the surface does not reflect the trophic status of the lake because most primary production is located at the DCM (Catalan et al., 2006). The lakes were selected in order to maximize variability in chemical and morphological characteristics and were accessed by foot as they are located in uninhabited remote locations.

SML samples were collected from the upper ~400 μm water with a nylon screen sampler (Agogué et al., 2004, 2005) near the deepest point of each lake. Surface (0.5 m depth) and deeper samples were taken using a 3-litre sampler (either Ruttner or Patalas bottles). Samples were pre-screened through a 40 μm pore-size net to remove large plankton components.

Water transparency was measured with a Secchi disk. Temperature profiles were measured *in situ* using a PT10 type thermistor. In the lab, pH was measured with an Orion instrument equipped with a probe for low ionic strength samples (Crison). Conductivity was measured with conductivimeter of the brand WTW. Total phosphorus (TP) was extracted with an oxidative persulfate digestion, and analyzed by spectrophotometry using the malachite green method (Camarero, 1994). Total nitrogen (TN) was extracted with an oxidative persulfate digestion in the autoclave, and measured by UV spectrometry. Dissolved organic carbon (DOC) was assessed on Whatman GF/F filtered lake-water with a Shimadzu TOC5000 analyzer. Chla concentrations were measured in 90% acetone extracts (1 L sample filtered on Whatman GF/F filter and extracted by 3 min sonication in 4 mL 90% acetone) according to Jeffrey and Humphrey (1975).

### Abundances of microbial food web components by flow cytometry

#### Sample collection

Microbial food web components (cell abundance of heterotrophic prokaryotes, PPP, and HNF) were quantified by flow cytometry. Three subsamples were taken for separate counts of heterotrophic prokaryotes, PPP, and heterotrophic flagellates. The last two samples were kept in water baths with *in situ* water and analyzed as soon as possible (3 h on average after collection). For heterotrophic prokaryotes counts, 4 ml of lake water were collected from each depth, fixed immediately with cold glutaraldehyde 10% (final concentration 1%), left in the dark for 10 min at *in situ* temperature, placed in liquid nitrogen and then stored at −80°C. Samples were analyzed 2 months after sampling. An aliquot of these frozen samples were used to quantify PPP and results were compared to the fresh analysis done *in situ* (see below).

#### Heterotrophic prokaryote abundances

Stored samples were thawed at room temperature and 400 μl were mixed with DMSO-diluted SYTO-13 (Molecular Probes Inc., Eugene, OR, USA) at 2.5 μmol l^−1^ final concentration. The mixture was left for about 10 min in the dark for complete staining and was run in a FACSCalibur (BectonDickinson) flow cytometer equipped with a 15 mW Argon-ion laser (488 nm emission). At least 30,000 events were acquired for each subsample. Heterotrophic prokaryotes were detected by their signature in a plot of side scatter (SSC) vs. FL1 (green fluorescence) following del Giorgio et al. (1996) as discussed in Gasol and del Giorgio (2000).

#### Phototrophic plankton abundances

The fixed samples were thawed and run without addition of any stain in a FACSCalibur flow cytometer equipped with a blue laser (488 nm, 15 mW) and a red laser diode (~635 nm). Small algae were identified in plots of SSC vs. FL3 (red fluorescence of Chla), FL2 (orange fluorescence of phycoerithrine, PE) vs FL3 (Olson et al., 1993), and FL3 vs. FL4 (far red fluorescence after red-light excitation, indicative of phycocyanine, PC). Samples were run twice with different settings (different voltages for scatter and fluorescence), in order to maximize the “sampling window” and cover cells sizes below 1 μm to ca. 20 μm, thus including pico- and nanophototrophs. Data analysis was performed with the CellQuest software (BectonDickinson), and we combined the information collected with the two settings into one set of populations. Examples of cytograms with distinct populations are presented in Schiaffino et al. (2013) and Sarmento et al. (2008). While the data presented in this study corresponds to samples analyzed a few months after collection and from fixed replicates, we also analyzed fresh samples the same day of collection, ~3 h after sampling. Since we did not have the red laser for these live samples, the numbers presented are those of the fixed sampled. The fresh samples were used to confirm that no populations were negatively affected by fixation and storage.

#### Small heterotrophic eukaryotes abundances

In this study, “small heterotrophic eukaryotes” included pico and nano eukaryotes, and refers mainly to small HNF. Small eukaryote abundances were estimated following the protocol by Rose et al. (2004). From a stock solution of 1 mM Lysotracker Green (Molecular Probes), 1 μl was added to 99 μl of <0.2 μm MilliQ, and 3.8 μl of this diluted Lysotracker were added to 0.5 ml of the sample, ending at 75 nM Lysotracker final concentration. We analyzed the samples as in Rose et al. (2004), using a combination of light scatter (SSC) and green (FL1) and red (FL3) fluorescence. Samples were run alive, less than 3 h after sample collection, and at high (ca. 100 μl min^−1^) speed. Concentrations were obtained from weight measurement of the volume analyzed.

### Bacterial bulk and single-cell activity

Bacterial bulk heterotrophic activity was estimated using the ^3^H-leucine incorporation method (Kirchman et al., 1985). Quadruplicate aliquots of 1.2 ml and 2 trichloroacetic acid (TCA) killed controls were taken immediately after sample collection. The samples were incubated with 40 nM ^3^H-leucine final concentration added immediately and for about 2 h in the dark in a water bath with lake water and at *in situ* temperature. The incorporation was stopped with the addition of 120 μl of cold TCA 50% to each replicate and the samples were kept frozen at –20°C until processing, which was carried out by the centrifugation method described by Smith and Azam (1992).

We used two physiological probes to test the metabolic and physiological single-cell status of prokaryotic microbes, respectively (i.e., Del Giorgio and Gasol, 2008). The abundance of respiring bacteria was determined using the probe 5-cyano-2,3-ditolyl tetrazolium chloride (CTC, Polysciences), an indicator of electron transport system respiratory activity. Highly respiring prokaryotes were considered those able to reduce CTC. Reduced CTC turns into a red fluorescent formazan that is detectable by epifluorescence and flow cytometry (Sherr et al., 1999; Sieracki et al., 1999). A stock solution of 50 mmol L^−1^ CTC (Polysciences) was prepared daily, filtered through 0.1 μm, and kept in the dark at 4°C until use. The CTC stock solution was then added to 0.45 mL of sample (5 mmol L^−1^ final CTC concentration) and incubated for 3 h at room temperature in the dark. The red fluorescence of CTC (FL3) and SSC were used to discriminate the CTC positive cells from other particles and an FL2-FL3 plot to exclude picoautotrophs. The percentage of CTC positive cells was calculated relative to the total bacterial counts obtained as mentioned above.

Cells with intact membranes were enumerated using the Nucleic-Acid-Double-Staining (NADS) viability protocol, which uses a combination of the cell-permanent nucleic acid strain SybrGreen I (SG1, Molecular Probes, Eugene, OR) and the cell-impermeant propidium iodine (PI, Sigma Chemical Co.) fluorescent probe. We used a 1:10 SG1 and 10 μg ml^−1^ PI concentrations that were added to fresh samples, less than 2 h after sampling. After simultaneous addition of each stain, the samples were incubated for 20 min in the dark at room temperature and then analyzed by flow cytometry. SG1 and PI fluorescence were detected in the green (FL1) and orange-red (FL3) cytometric channels, respectively. A dot plot of red vs. green fluorescence allowed distinction of the “live” cells (i.e., cells with intact membranes and DNA present) from the “dead” cells (i.e., with compromised membranes, (Grégori et al., 2001; Falcioni et al., 2008).

### Statistical analyses

We computed a neuston enrichment factor as the ratio between abundance or activity in the SML divided by values measured at the surface to quantify the magnitude of the differences between layers. We tested for differences between layers using paired Student's *t*-tests. In order to explore possible relationships between environmental variables and microbial abundance or activity obtained for the different lakes and lake layers, we run a Factor Analysis (FA) using SPSS 20 Statistics and the stats package in R software (R Core Team, 2014). We separated physical and chemical variables and performed two different FA for each set of environmental variables. We included lake and catchment areas, altitude, maximum depth, surface temperature, and Secchi depth as physical variables. As for chemical variables, we included alkalinity, conductivity, DOC, total dissolved phosphorus (TDP), NH^+^_4_, NO^−^_2_, NO^−^_3_, SO^2−^_4_, Ca^2+^-dissolved reactive silica (DRSi), Chla. We had measured other parameters (pH, soluble reactive phosphorus, total dissolved nitrogen, DIC, Cl^−^, Na^+^, K^+^, Mg^2+^) that were excluded from the FA because they were highly correlated or were a lineal combination of some of the parameters included and violated the requirement of uniqueness. All variables were previously log transformed to normalize their distribution. The Kolmogorov–Smirnoff test was applied to check that there were no strong departures from normality. The abundance or activity variables at the surface and the SML, and the enrichment factor were tested for correlation against the scores obtained for the physical and chemical factors.

## Results

### *In situ* physical and chemical conditions

The formation of a true SML requires stable conditions of wind and temperature for a certain amount of time. We sampled 19 lakes in a window of 9 days in which stable and sunny conditions occurred in the mountain. In order to detect the presence of a true SML, we first scanned our database, comparing temperature in the first millimeters of surface water (temperature profiles in Supplementary Material). We considered that there was no true SML in lakes in which water temperature was equal (at the 0.01°C resolution, the smallest reliable according to the resolution of the temperature probe used) in the lake skin and few millimeters below the surface, and we discarded these lakes from the analysis (indicated with “∗” in Table 1), by considering that a true SML did not exist in those lakes. From here on, we refer only to the results obtained in the 16 selected lakes, which developed a true SML at the moment they were sampled.

**Table 1:** **Location, general features, and some limnological parameters of the 21 samples from Pyrenean high-altitude lakes (DOC, dissolved organic carbon; TP, total phosphorous; TN, total nitrogen)**.

**Lake**	**Latitude**	**Longitude**	**Altitude (m asl)**	**Max. depth (m)**	**pH units**	**Conductivity (mS cm^−1^)**	**DOC (mg l^−1^)**	**TP (nM)**	**TN (μM)**	**Chl *a* (μg l^−1^)**	**Secchi depth (m)**
^*^Aixeus	42°36′40″	1°22′18″	2370	15.5	4.97	49.9	0.2	74	18	0.6	15.5
^*^Bassa de les Granotes	42°34′24″	0°58′16″	2330	5	6.45	9.8	2.3	119	26	4.6	2.4
Botornàs	42°35′35″	0°40′52″	2340	22	7.23	24.1	0.3	168	26	2.0	12.4
Certascan	42°42′40″	1°18′12″	2335	113	5.67	9.1	0.2	78	35	0.2	19.6
Filià	42°27′4″	0°57′12″	2140	5.5	7.79	133.3	not available	94	21	1.7	1.3
Gerber	42°37′50″	0°59′41″	2170	63	7.13	23.4	0.6	88	9	1.3	11.8
Ibonet Perramó	42°38′34″	0°29′49″	2293	5	7.49	33.3	0.4	150	17	0.6	5.2
Illa	42°37′6″	0°59′37″	2452	18	6.68	13.3	0.4	70	7	1.3	12.0
Llauset	42°39′22″	0°41′11″	2190	90	7.52	58.7	not available	113	18	1.1	10.0
Llebreta	42°33′3″	0°53′25″	1620	11.5	7.55	30.6	1.1	203	not available	1.8	7.9
Long de Liat	42°48′24″	0°52′26″	2140	32	7.27	20.8	0.7	68	5	1.0	11.8
Muntanyó d′Àrreu	42°40′26″	1°00′29″	2210	29	9.18	74.3	0.8	674	9	12.0	10.1
Pica Palomèra	42°47′38″	0°52′8″	2308	10	4.61	29.6	not available	50	9	0.5	8.0
Plan	42°37′21″	0°55′51″	2188	11	7.04	16.7	1.4	63	7	0.6	9.0
Pòdo	42°36′11″	0°56′21″	2450	20	6.41	9.4	0.3	273	79	4.2	9.6
Pois	42°39′20″	0°42′27″	2056	19.5	8.19	67.2	0.2	82	14	2.9	8.6
^*^Redon	42°38′33″	0°46′13″	2240	73	6.67	10.5	0.4	n.a.	n.a.	1.5	n.a.
Roi	42°34′29″	0°48′12″	2310	10	7.06	25.8	0.5	126	18	5.2	5.9
Romedo de Dalt	42°42′22″	1°19′29″	2114	40	6.31	5.8	0.8	103	13	2.8	14.4

Chemical and Chla values measured at the depth of the deep chlorophyll maxima. (

* *Lakes discarded from the analysis, see text)*.

Most of the lakes sampled were located at >2000 m above sea level and can be considered as high altitude lakes. This set of lakes includes a large range of maximal depths (from 5 to 113 m) and geochemical types. As an example, the pH among the 16 lakes ranged from 4.6 to 9.2, and conductivity ranged from 6 to 133 μS cm^−1^ (Table 1). All the lakes were oligotrophic except Muntanyó d'Àrreu, which was mesotrophic (Table 1). Average daily-integrated long-wave solar radiation during the sampling period (17th–24th July 2008) was 26.4 ± 8.3 MJ m^−2^. The UV doses corresponding to this radiation were 1.4 ± 0.4 kJ m^−2^ at 305 nm, 8.2 ± 2.5 kJ m^−2^ at 320 nm, 15.3 ± 4.5 kJ m^−2^ at 340 nm, and 20.7 ± 6.3 kJ m^−2^ at 380 nm.

Chla (measured at the DCM) ranged from 0.2 μg l^−1^ in Lake Certascan to 12.0 μg l^−1^ in Lake Muntanyó d'Àrreu. Accordingly, water transparency ranged between 1.3 and 19.6 m of Secchi disk depth. Chla was correlated to total phosphorus (*n* = 17, *p* < 0.001, *r*^2^ = 0.70) and nitrogen (*n* = 17, *p* < 0.01, *r*^2^ = 0.58). Dissolved organic carbon (DOC) was relatively low, ranging from 0.2 to 1.4 mg l^−1^ in Lake Certascan and Lake Plan, respectively.

### Abundances of microbial food web components

#### Phototrophic components

**Figure 1:**
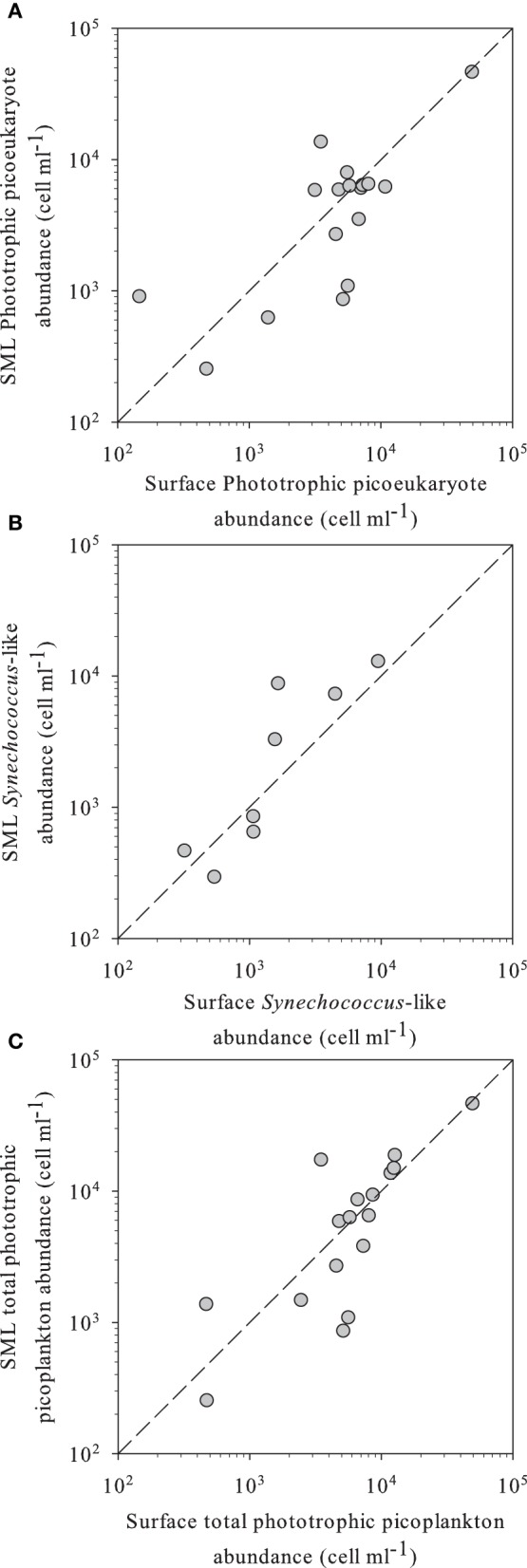
**(A)** Phototrophic small eukaryotes, **(B)** *Synechococcus*-like, and **(C)** total phototrophic picoplankton abundance in SML vs. surface waters of the different high altitude lakes studied, as determined by flow cytometry. In all panels, the dashed line indicates the 1:1 ratio.

Flow cytometry-determined abundances of PPP ranged two orders of magnitude from 2.5 × 10^2^ to 1.8 × 10^4^ ml^−1^, with slightly higher average abundances in the SML than in the surface waters, although not significant (Table 2). We observed a high diversity of populations in the cytograms obtained. The number of identified populations was on average 4.1 (range 1–7). In general we identified higher diversity of small phototrophic eukaryotes (red-fluorescing cells) than of phycoerythrin (PE)-rich (picocyanobacteria). None of the considered lakes had red-fluorescing (phycocyanin-rich) cyanobacteria. While all lakes had small phototrophic eukaryotes, some did not have cyanobacteria. In lakes with no populations of cyanobacteria, total PPP abundances were higher in the surface than in the SML. Lakes harboring PE-rich populations had higher total PPP abundance in the SML. Moreover, PE-rich (*Synechococcus*-like) cells were more abundant in the SML than in the surface, in those lakes where such populations were present (Figure 1). In most lakes, the number of different populations observed by flow cytometry (cytometric diversity) was higher at the surface than in the SML (Table 2). In a general comparison, the diversity of populations was higher at the surface but abundances were higher at the SML, although most comparisons were not significant or at the significance borderline (Table 2).

**Table 2:** **Average and range of values of the different variables measured in the SML and surface plankton of the studied lakes**.

**Variable**	**SML**	**Surface**	***P* (paired *t*-test)**
**PHOTOTROPHIC PICOPLANKTON**			
Total PPP abundance	7.1 10^3^ (0.3−18.9)	6.3 10^3^ (0.5−12.6)	NS
Picocyanobacteria	2.4 10^3^ (0−13.0)	1.3 10^3^ (0−9.5)	0.02
Small eukaryotes	4.7 10^3^ (0.3−13.7)	5.0 10^3^ (0.2−10.8)	NS
Ratio small eukaryotes:cyanobacteria	150 (0-650)	238 (0−800)	0.03
Number of diff. PPP populations	4.1 (1−7)	4.5 (2−7)	NS
Number of diff. picocyanobacterial pop.	0.8 (0−2)	0.6 (0−2)	NS
Number of diff. small eukaryotes populations	3.3 (1−5)	3.9 (2−6)	0.04
**HETEROTROPHIC PICOPLANKTON**			
Total heterotrophic prokaryotes	1.0 10^6^ (0.3−2.0)	0.8 10^6^ (0.3−1.8)	0.03
NADS-Live prokaryotes	5.0 10^5^ (0.7−9.9)	4.9 10^5^ (1.1−12.1)	NS
NADS-Dead prokaryotes	5.1 10^5^ (1.8−11.2)	3.2 10^5^ (0.8−5.4)	0.01
HNF abundance	3.0 10^3^ (0.9−8.8)	2.0 10^3^ (0.9−5.7)	0.02
Ratio Total heterotrophic prokaryotes:HNF	482 (63−1297)	491 (135−1122)	NS
CTC positive prokaryotes	3.7 10^4^ (2.1−6.1)	2.8 10^4^ (1.6−5.8)	0.01
Prokaryotic heterotrophic act. (leucine inc.)	9.5 (1.4−29.0)	27.9 (7.4−61.4)	<0.01
Cell-specific prokaryotic activity	9.9 (3−31)	36.0 (16−64)	<0.01
% Live prokaryotes	49 (22−70)	59 (44−82)	<0.01

*Abundances in cells ml^−1^. Bacterial activity in pmol Leu ml^−1^ h^−1^ and cell-specific activity in amol cell^−1^ h^−1^. Averages and the statistical comparison of the picophytoplankton (PPP) abundance data do not include Bassa de les Granotes, an obvious outlier. All variables were log transformed to achieve normality prior to the paired t-test*.

#### Heterotrophic components

**Figure 2:**
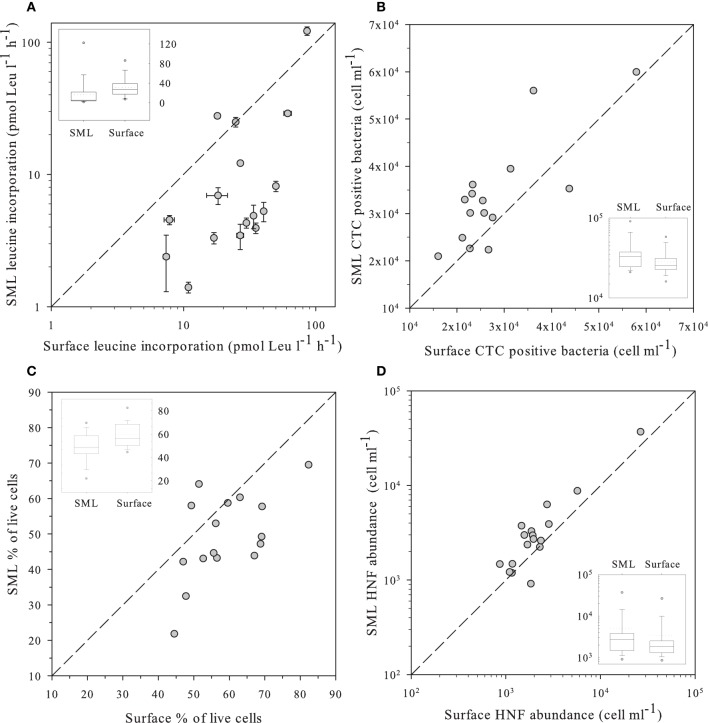
**(A)** Leucine incorporation in SML samples vs. values observed in the surface; **(B)** Number of heterotrophic prokaryote cells with high respiration rates (CTC+) in SML samples vs. those observed in the surface as determined by flow cytometry and the CTC staining protocol. **(C)** Percentage of “live” cells in the SML samples vs. the value observed at the surface as determined by flow cytometry and the NADS staining protocol. **(D)** Abundance of HNF in SML samples vs. values observed in the surface measured by flow cytometry with the Lysotracker staining protocol. In all panels, the dashed line indicates the 1:1 ratio. Insets in each panel indicate the distribution of values for surface and the SML. The central line indicates the median value, boxes indicate the lower and upper quartiles, and vertical lines indicate the 10th and 90th percentiles.

We observed significantly more heterotrophic prokaryotes and HNF in the SML than in surface waters (Table 2, Figure 2D). The total heterotrophic prokaryote:HNF ratio was relatively low (~480–490 bacteria per HNF, Table 2), and no significant differences were found between layers.

### Bulk and single-cell prokaryote heterotrophic activities

We measured several indices of bacterial activity, some at the bulk level, such as leucine incorporation, and others at the single-cell level, such as abundance of bacterial cells presenting high levels of respiration (i.e., CTC positive). We used plots of surface against SML samples for the different parameters to discriminate the variables that did not randomly lay around the 1:1 ratio (Figure 2).

Leucine incorporation varied in a large range, from 1.4 to 121 pmol Leu l^−1^ d^−1^ and was systematically higher in the surface than in the SML with the exception of lakes Pois and Roi (Figure 2A). A similar plot for CTC indicated that the number of heterotrophic prokaryotic cells with high respiration rates was higher in the SML than at the surface, as most values were above the 1:1 ratio (Figure 2B), Overall, there were more respiring prokaryotic cells at the SML, while they produced more in surface waters, both trends being highly significant (Table 2).

The NADS staining protocol (that measures the relationships between membrane-intact and membrane-damaged heterotrophic prokaryotic cells) showed healthier heterotrophic prokaryotes in surface plankton than at the neuston (Figure 2C). In fact, damaged cells accumulated at the SML while intact cells had similar abundances in both layers (Table 2).

### Enrichment factors

**Figure 3:**
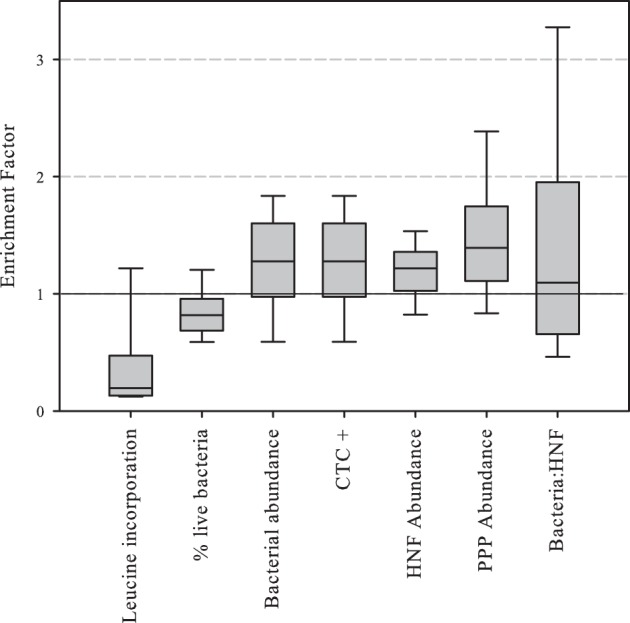
**Boxplot of abundance or activity enrichment factors in the piconeuston (i.e., SML:surface)**. Central line indicates median value, boxes the lower and upper quartiles, and vertical lines indicate 10th and 90th percentiles.

Enrichment factors were calculated as the ratio between the values of abundance or activity in the SML divided by those in surface waters. The results of enrichment factors indicated different degrees of enrichment in the neuston for CTC positive, and bacterial, HNF and PPP abundances. On the contrary, leucine incorporation and the percentage of “live” bacterial cells were lower in the neuston (Figure 3).

### Factor analysis

FA simplifies the interpretation of the many environmental variables measured in the 16 lakes. We performed separated FA for physical and chemical variables, and the three first factors explained 85.3–64.5% of the variance, respectively (Table 3). The first three physical factors reflected lake morphometry (Factor F1), altitude (Factor F2), and water temperature (Factor F3). Regarding chemical factors, the FA analysis revealed productivity (Factor C1) as the main factor, then DOC, silica, sulfate, and nitrate concentration (Factor C2), and finally conductivity and calcium concentration (Factor C3), which would capture the lithology as reflected in water chemistry. A fourth factor was obtained on which NH^+^_4_ (negatively) and NO^−^_2_ and alkalinity (positively) loaded. However, this factor was forced by a single outlier lake. It did not show any correlation with any biological parameter and therefore it is not discussed here. Most factors were easily interpretable with exception of factor C2 (DOC, silica, sulfate, and nitrate). In this factor, DOC, silica, and sulfate were the strongest variables (higher loadings), and nitrate, to a lesser extend. Silica and sulfate are usually related to the metamorphic nature of rocks in the Pyrenees (Catalan et al., 1993). This factor C2 could be interpreted as a “watershed DOC factor,” representing a gradient from a rockier watershed with low DOC production (high silica and sulfate from metamorphic rock weathering), to higher soil coverage with higher DOC production (less silica and sulfate). An alternative explanation is that the covariation of these three parameters could be related to the landscape position of the lakes (Sadro et al., 2011).

**Table 3:** **Factor loadings and cumulative variance explained obtained in the factor analysis of chemical and physical variables of the 17 high altitude lakes studied**.

**Physical variables**	**Factor F1**	**Factor F2**	**Factor F3**
	**Lake morphometry**	**–Altitude**	**–Temperature**
Zmax	0.946		
Lake area	0.874		
Secchi depth	0.685		
Watershed area		0.926	
Altitude		−0.884	
Water temperature			−0.911
Cumulative Variance	37.8%	67.4%	85.3%
**Chemical variables**	**Factor C1**	**Factor C2**	**Factor C3**
	**Productivity**	**–Watershed DOC**	**Lithology**
TP	0.922		
Chla	0.776		
DOC		−0.769	
DRSi		0.765	
SO_4_		0.678	
NO_3_		0.526	
Conductivity			0.951
Ca2+			0.937
Cumulative Variance	21.8%	43.4%	64.5%

We tested for correlation of the scores of the physical factors against those of the chemical factors and no significant correlations were found, indicating that the factors were independent (and so were the variables that laid behind them). One exception was the correlation between factors F3 (water temperature) and C2 (watershed DOC), which were positively correlated (*p* < 0.05, Pearson coefficient = 0.518).

In a second step, the abundance or activity variables in the two layers (surface and SML), and the enrichment factors were tested for correlation against the scores of the main chemical and physical factors (Table 4). In general, biological variables in the SML were more related to physical factors, especially lake morphometry (Factor F1) and temperature (Factor F3) and to the nature of the rock (Factor C3). Conversely, surface biological variables were frequently correlated with the Factor C2 (watershed DOC), but also with water temperature (Factor F3). Concerning enrichment factors, the same pattern was observed: stronger and more correlations were found with the Factor C2 (watershed DOC).

**Table 4:** Pearson correlation coefficient (r) of the statistically significant correlations (^*^p < 0.05 or ^**^p < 0.01) between the variables measured in the 17 high altitude lakes and the chemical and physical scores (obtained in the factor analyses, see Table 3).

	**Factor F1**	**Factor F2**	**Factor F3**	**Factor C1**	**Factor C2**	**Factor C3**
	**Lake morphometry**	**–Altitude**	**–Temperature**	**Productivity**	**–Watershed DOC**	**Lithology**
**SML**						
Total heterotrophic prokaryotes	−0.654^**^					0.558^*^
% Live prokaryotes	−0.536^*^		−0.523^*^			
Total PPP abundance			−0.652^**^			
Prokaryotic heterotrophic activity (leucine incorporation)		0.540^*^				
**SURFACE**						
% Live prokaryotes	−0.498^*^					
CTC positive prokaryotes		0.535^*^				
Ratio Total heterotrophic prokaryotes:HNF		−0.531^*^			0.488^*^	−0.544^*^
Total heterotrophic prokaryotes			−0.628^**^		−0.533^*^	
Total PPP abundance				0.530^*^	−0.540^*^	
Prokaryotic heterotrophic activity (leucine incorporation)					−0.567^*^	
**ENRICHMENT FACTOR**						
Total PPP abundance			−0.506^*^			
Ratio Total heterotrophic prokaryotes:HNF				0.677^**^		
Prokaryotic heterotrophic activity (leucine incorporation)					0.713^**^	

## Discussion

Consistent differences were observed for picoplankton abundance, activity, and single cell physiological status between the SML and surface waters for the great majority of the 16 high mountain lakes that had developed a SML. In most lakes, PPP abundances were higher in the SML, particularly when PE-rich populations were present, whereas leucine incorporation rates in the SML were clearly below the values found in underlying waters, and these differences were probably related to the *in situ* stressful environmental conditions experienced by the neuston.

All the lakes studied had several PPP populations, and roughly half of them had picocyanobacteria. As far as we are aware, no other study had reported such a widespread presence of PPP in this type of environments, i.e., lakes located above 2000 m, although a large genetic diversity of planktonic eukaryotic microorganisms (3–40 microns sized) has been recently reported from the same Pyrenean lakes dataset (Triadó-Margarit and Casamayor, 2012).

Concerning heterotrophic prokaryotes, leucine incorporation was clearly lower in the SML compared to surface waters. A possible explanation for this observation is the direct effect of sunlight, particularly UVR, on the prokaryotic community. Several studies have demonstrated the inhibitory effect of solar radiation on substrate (thymidine, leucine, glucose, and acetate) incorporation rates on exposed prokaryotic communities, compared to dark or UVR-free incubations (Herndl et al., 1993; Sommaruga et al., 1997; Santos et al., 2012b; Ruiz-González et al., 2013). Actually, these processes are known to be by far more complex, as different bacterial groups have different sensitivities to UVR exposure, some of them being strongly inhibited (such as the SAR11 clade, at least in marine systems), some resistant (such as *Gammaproteobacteria* and *Bacteroidetes*), and some stimulated (such as *Roseobacter*) by natural sunlight (Alonso-Sáez et al., 2006; Santos et al., 2012a). The observed bulk community response could therefore be the sum of the positive and negative effects on the different prokaryotic populations.

The theoretical framework outlined by Carlson et al. (2007) postulates that increased environmental hostility (lack of resources - organic matter or nutrients, salinity, extreme temperature, UVR, extreme pH values, or a combination of several of these) should result in an increase in the proportion of the total flux of energy that is devoted to cell maintenance. Associated with this increase in cell maintenance, cell-specific respiration should increase to fuel the increased maintenance and the repair mechanisms. Thus, bacterial growth efficiency (BGE) would be expected to decrease with increasing environmental hostility. Our results provide experimental evidence that validate this hypothesis, at least in high-mountain lakes with high levels of UVR: not only bulk bacterial activity was lower in the SML (ca. four times lower on average), but the number of heterotrophic prokaryote cells with high respiration rates (CTC positives) was higher in the SML than at the surface (1.5x). We also observed a higher percentage of membrane-damaged heterotrophic prokaryotes in the SML than in the surface, probably as a consequence of the postulated environmental hostility. The number of CTC-positive cells has been found to correlate with total bacterial respiration in several previous studies (Cook and Garland, 1997; Smith, 1998; Berman et al., 2001).

It is unlikely that virus-induced lysogeny could be the main cause for the differences observed between layers, as it is known that solar radiation, particularly UV-B radiation (290–320 nm), is an important factor contributing to the decay of aquatic viruses (Suttle and Chen, 1992). Moreover, a previous study in a high mountain lake reported a negative correlation between viral abundances and solar radiation (Hofer and Sommaruga, 2001). In addition to HNF pressure, higher number of membrane-damaged heterotrophic prokaryotes cells in the SML comparing to surface, can also be explained by the fact that many airborne bacteria can be trapped in the air-water interface (Hervàs and Casamayor, 2009).

Our observations contrast with a recent study in 6 alpine lakes situated in an altitude gradient, which could not find a consistent pattern in the comparison of bacterial production between layers, and attributed the variation observed to lake-specific communities and environmental conditions (Hörtnagl et al., 2010). However, in such study, 3 out of 4 lakes located above 1500 m a.s.l. had lower bacterial production values in the SML than at the surface. In our study, most lakes were above 2000 m a.s.l.. As UVR increases with altitude (Blumthaler et al., 1997), this might explain the discrepancies between our study and that of Hörtnagl et al. Additionally, in our case, we only sampled under very stable weather conditions, sunny and calm, something that might have facilitated the detection of a well-formed SML with a microbiota strongly affected by solar radiation.

To the best of our knowledge, this is the first report of HNF abundance in freshwater SML and our results show that the neuston holds generally more HNF than surface lake water. The enrichment factor of HNF in the SML was, in average, slightly higher (1.47) than that of bacteria (1.27). These observations are somehow unexpected, as previous studies had shown that UVR affects negatively HNF growth and grazing on bacteria (Sommaruga et al., 1999). The total heterotrophic prokaryote:HNF ratio might provide some hints on this issue, and although not significantly different, this ratio was slightly higher in the SML. In addition, absolute values (~480–490 bacteria per HNF) were relatively low compared to literature data (Gasol, 1994; Fermani et al., 2013). This might indicate relatively high grazing pressure of HNF on bacteria, which might have to do with specific features of food web structure in high altitude lakes. *Daphnia*, and large cladocerans in general, are particularly sensitive to UVR reviewed by Sommaruga (2001). The dominance of large filter feeding cladocerans reduces HNF abundance, and affects the microbial food web (Gasol et al., 1995; Zöllner et al., 2003; Sarmento, 2012). Thus, HNF from high altitude lakes are likely to undergo low predation pressure, and this would result in higher grazing pressure on bacteria, providing an explanation for the relatively low heterotrophic prokaryote:HNF ratio observed in this study.

No simple and direct linear relationships could be found between microbial abundances or activities and raw environmental variables. Nevertheless, factors derived from the FA did correlate in some cases with biological variables. Both physical and chemical FA captured a fairly good percentage of the total variance (85.3–64.5% respectively), which means that the extracted factors successfully explained most of the variability observed in our dataset. It has to be noted that our data do not account of course for all aspects of environmental variability. Our discussion includes only those aspects captured by our measurements, and further research may seed light on other aspects not considered here.

The physical and chemical multivariate factors identified in the FA were not correlated between them, reinforcing the independency of the factors, with the exception of factors F3 (–temperature) and C2 (–watershed DOC), which were positively correlated (*p* < 0.05, *r* = 0.518). It is possible that higher temperatures promote higher DOC production in the watershed increasing allochthonous DOC inputs to the water. Two alternative hypotheses are that lakes with higher DOC are warmer because of solar radiation absorption, or that snowmelt increases stream DOC inputs into lakes.

Interestingly, the factors that influenced heterotrophic prokaryote abundance in the different layers were different. On one hand, prokaryote abundance in SML was negatively correlated with lake morphometry (factor F1). On the other hand, surface abundance was negatively correlated with factor F3 (–temperature). Larger lakes have greater fetch and turbulence, and probably present less well-defined SML and present them less often than smaller lakes. Larger lakes might thus lack some taxa well-adapted to SML conditions (environmental filtering), while surface prokaryote abundance depends on water temperature, regardless of the size of the lake.

Prokaryote abundance and activity in the surface co-varied with watershed DOC (factor C1) but not in the SML (Table 4). This suggests that surface and piconeuston microbial communities rely on different energy and/or nutrient sources. Pyrenean high mountain lakes receive considerable amounts of atmospheric deposition, namely from the Sahara desert (Mladenov et al., 2011; Barberán et al., 2014) that might be the main source of nutrients and organic matter for the piconeuston.

Another interesting outcome of the FA is the fact that altitude (factor F2) correlated negatively with prokaryotic activity in the SML. In other words, leucine incorporation in the SML decreased with altitude. UVR increases with altitude, but part of the radiation is reflected and absorbed in the first millimeters of the water column, and the UVR that reaches subsurface water is not as high as that reaching the SML (Sommaruga and Psenner, 1997), This probably explains why leucine incorporation in subsurface waters had no relationship with altitude. However, in the SML exposure levels are of such magnitude that anabolic processes are affected according to the UVR dose (i.e., directly correlated to altitude).

Finally, the prokaryotic activity enrichment factor (which was always lower than one, Figure 3) was correlated to factor C2 (–watershed DOC), meaning that in rocky watersheds with low DOC production, neuston and surface prokaryotic activity had relatively small differences, and these differences increased in DOC enriched watersheds. Basically, prokaryotic activity increased in DOC-rich surface waters, but not in neuston, indicating that despite the physical proximity between these two habitats, the processes shaping microbial metabolism were substantially different.

In conclusion, we found evidence that heterotrophic prokaryotic communities in the piconeuston of high altitude lakes had lower percentage of healthy cells, lower leucine incorporation rates and higher number of cells with high respiration rates (CTC positive), likely resulting in lower BGE as compared to those of the underlying surface water, evidencing a high degree of environmental harshness. PPP were widespread in these high mountain lakes and PE-rich picocyanobacteria were particularly prominent in the SML. Piconeuston was also enriched in HNF and heterotrophic prokaryotes. Overall, we demonstrate that steep gradients in the upper millimeters of surface waters of high mountain lakes are common in stable conditions, and that different mechanisms drive microbial processes in the SML and the underlying surface water.

### Conflict of interest statement

The authors declare that the research was conducted in the absence of any commercial or financial relationships that could be construed as a potential conflict of interest.

## Supplementary material

Detailed temperature profiles in the high mountain lakes studied in the sampling day.

## References

[B1] Agogué H. Casamayor E. O. Bourrain M. Obernosterer I. Joux F. Herndl G. J.. (2005). A survey on bacteria inhabiting the sea surface microlayer of coastal ecosystems. FEMS Microbiol. Ecol. 54, 269–280. 10.1016/j.femsec.2005.04.002 16332325

[B2] Agogué H. Casamayor E. O. Joux F. Obernosterer I. Dupuy C. Lantoine F. (2004). Comparison of samplers for the biological characterization of the sea surface microlayer. Limnol. Oceanogr. Methods 2, 213–225 10.4319/lom.2004.2.213

[B3] Alonso-Sáez L. Gasol J. M. Lefort T. Hofer J. Sommaruga R. (2006). Effect of natural sunlight on bacterial activity and differential sensitivity of natural bacterioplankton groups in northwestern Mediterranean coastal waters. Appl. Environ. Microbiol. 72, 5806–5813. 10.1128/AEM.00597-06 16957198 PMC1563624

[B4] Auguet J. C. Casamayor E. O. (2008). A hotspot for cold crenarchaeota in the neuston of high mountain lakes. Environ. Microbiol. 10, 1080–1086. 10.1111/j.1462-2920.2007.01498.x 18215160

[B5] Barberán A. Henley J. Fierer N. Casamayor E. O. (2014). Structure, inter-annual recurrence, and global-scale connectivity of airborne microbial communities. Science Total Environ. 487, 187–195. 10.1016/j.scitotenv.2014.04.030 24784743

[B6] Berman T. Kaplan B. Chava S. Viner Y. Sherr B. F. Sherr E. B. (2001). Metabolically active bacteria in Lake Kinneret. Aquat. Microb. Ecol. 23, 213–224 10.3354/ame023213

[B7] Blumthaler M. Ambach W. Ellinger R. (1997). Increase in solar UV radiation with altitude. J. Photochem. Photobiol. B Biol. 39, 130–134 10.1016/S1011-1344(96)00018-8

[B8] Camarero L. (1994). Assay of soluble reactive phosphorus at nanomolar levels in nonsaline waters. Limnol. Oceanogr. 39, 707–711 10.4319/lo.1994.39.3.0707

[B9] Carlson C. A. del Giorgio P. A. Herndl G. J. (2007). Microbes and the dissipation of energy and respiration: from cells to ecosystems. Oceanography 20, 89–100 10.5670/oceanog.2007.52

[B10] Catalan J. Ballesteros E. Gacia E. Palau A. Camarero L. (1993). Chemical composition of disturbed and undisturbed high-mountain lakes in the Pyrenees: a reference for acidified sites. Water Res. 27, 133–141 10.1016/0043-1354(93)90203-T

[B11] Catalan J. Camarero L. Felip M. Pla S. Ventura M. Buchaca T. (2006). High mountain lakes: extreme habitats and witnesses of environmental change. Limnetica 25, 551–584.

[B12] Conrad R. Seiler W. (1988). Influence of the surface microlayer on the flux of nonconservative trace gases (CO, H_2_, CH_4_, N_2_O) across the ocean-atmosphere interface. J. Atmos. Chem. 6, 83–94 10.1007/BF00048333

[B13] Cook K. L. Garland J. L. (1997). The relationship between electron transport activity as measured by CTC Reduction and CO2 production in mixed microbial communities. Microb. Ecol. 34, 237–247. 10.1007/s002489900053 9337419

[B14] Cunliffe M. Upstill-Goddard R. C. Murrell J. C. (2011). Microbiology of aquatic surface microlayers. FEMS Microbiol. Rev. 35, 233–246. 10.1111/j.1574-6976.2010.00246.x 20726895

[B14a] Del Giorgio P. A. Gasol J. M. (2008). Physiological structure and single-cell activity in marine bacterioplankton, in Microbial Ecology of the Oceans, 2nd Edn., ed Kirchman D. L. (New York, NY: John Wiley & Sons, Inc.), 243–298 10.1002/9780470281840.ch8

[B15] del Giorgio P. A. Gasol J. M. Vaqué D. Mura P. Agustí S. Duarte C. M. (1996). Bacterioplankton community structure: protists control net production and the proportion of active bacteria in a coastal marine community. Limnol. Oceanogr. 41, 1169–1179 10.4319/lo.1996.41.6.1169

[B16] Dietz A. S. Albright L. J. Tuominen T. (1976). Heterotrophic activities of bacterioneuston and bacterioplankton. Can. J. Microbiol. 22, 1699–1709. 10.1139/m76-251 1009500

[B17] Falcioni T. Papa S. Gasol J. M. (2008). Evaluating the flow-cytometric nucleic acid double-staining protocol in realistic situations of planktonic bacterial death. Appl. Environ. Microbiol. 74, 1767–1779. 10.1128/AEM.01668-07 18223113 PMC2268295

[B18] Fermani P. Diovisalvi N. Torremorell A. Lagomarsino L. Zagarese H. Unrein F. (2013). The microbial food web structure of a hypertrophic warm-temperate shallow lake, as affected by contrasting zooplankton assemblages. Hydrobiologia 714, 115–130 10.1007/s10750-013-1528-3

[B19] Gasol J. M. del Giorgio P. A. (2000). Using flow cytometry for counting natural planktonic bacteria and understanding the structure of planktonic bacterial communities. Sci. Mar. 64, 197–224 10.3989/scimar.2000.64n2197

[B20] Gasol J. M. Simons A. M. Kalff J. (1995). Patterns in the top-down versus bottom-up regulation of heterotrophic nanoflagellates in temperate lakes. J. Plankton Res. 17, 1879–1903 10.1093/plankt/17.10.1879

[B21] Gasol J. M. (1994). A framework for the assessment of top-down vs bottom-up control of heterotrophic nanoflagellate abundance. Mar. Ecol. Prog. Ser. 113, 291–300 10.3354/meps113291

[B22] Grégori G. Citterio S. Ghiani A. Labra M. Sgorbati S. Brown S.. (2001). Resolution of viable and membrane-compromised bacteria in freshwater and marine waters based on analytical flow cytometry and nucleic acid double staining. Appl. Environ. Microbiol. 67, 4662–4670. 10.1128/AEM.67.10.4662-4670.2001 11571170 PMC93217

[B23] Herndl G. J. Muller-Niklas G. Frick J. (1993). Major role of ultraviolet-B in controlling bacterioplankton growth in the surface layer of the ocean. Nature 361, 717–719 10.1038/361717a0

[B24] Hervàs A. Casamayor E. O. (2009). High similarity between bacterioneuston and airborne bacterial community compositions in a high mountain lake area. FEMS Microbiol. Ecol. 67, 219–228. 10.1111/j.1574-6941.2008.00617.x 19049500

[B25] Hofer J. S. Sommaruga R. (2001). Seasonal dynamics of viruses in an alpine lake: Importance of filamentous forms. Aquat. Microb. Ecol. 26, 1–11 10.3354/ame026001

[B26] Hörtnagl P. Perez M. T. Sommaruga R. (2010). Living at the border: a community and single-cell assessment of lake bacterioneuston activity. Limnol. Oceanogr. 55, 1134–1144. 10.4319/lo.2010.55.3.1134 20401318 PMC2981156

[B27] Jeffrey S. W. Humphrey G. F. (1975). New spectrophotometric equations for determining chlorophylls a1, b1, c1 and c2 in higher plants, algae and natural phytoplankton. Biochem. Physiol. Pflanz. 167, 194–204.

[B28] Kirchman D. K'nees E. Hodson R. (1985). Leucine incorporation and its potential as a measure of protein synthesis by bacteria in natural aquatic systems. Appl. Environ. Microbiol. 49, 599–607. 3994368 10.1128/aem.49.3.599-607.1985 PMC373556

[B29] Kuznetsova M. Lee C. (2001). Enhanced extracellular enzymatic peptide hydrolysis in the sea-surface microlayer. Mar. Chem. 73, 319–332 10.1016/S0304-4203(00)00116-X

[B30] Kuznetsova M. Lee C. Aller J. Frew N. (2004). Enrichment of amino acids in the sea surface microlayer at coastal and open ocean sites in the North Atlantic Ocean. Limnol. Oceanogr. 49, 1605–1619 10.4319/lo.2004.49.5.1605

[B31] Maki J. S. (1993). The air-water interface as an extreme environment, in Aquatic Microbiology: An Ecological Approach, ed Ford T. E. (Boston; Oxford: Blackwell Scientific Publications), 409–439.

[B32] Mladenov N. Sommaruga R. Morales-Baquero R. Laurion I. Camarero L. Dieguez M. C.. (2011). Dust inputs and bacteria influence dissolved organic matter in clear alpine lakes. Nat. Commun. 2:405. 10.1038/ncomms1411 21792184 PMC3144587

[B33] Naumann E. (1917). Beiträge zur kenntnis des teichnanoplanktons. II. Über das neuston des süßwasser. Biologisches Zentralblatt 37, 98–106.

[B34] Olson R. J. Zettler E. R. Du Rand M. D. (1993). Phytoplankton analysis using flow cytometry, in Handbook of Methods in Aquatic Microbial Ecology, eds Kemp P. F. Sherr B. F. Sherr E. B. Cole J. J. (Boca Raton FL: Lewis Publishers), 175–186.

[B35] R Core Team (2014). R: A Language and Environment for Statistical Computing. Foundation for Statistical Computing, Vienna, Austria Available online at: http://www.R-project.org/

[B36] Rose J. M. Caron D. A. Sieracki M. E. Poulton N. (2004). Counting heterotrophic nanoplanktonic protists in cultures and aquatic communities by flow cytometry. Aquat. Microb. Ecol. 34, 263–277 10.3354/ame034263

[B37] Ruiz-González C. Simó R. Sommaruga R. Gasol J. M. (2013). Away from darkness: a review on the effects of solar radiation on heterotrophic bacterioplankton activity. Front. Microbiol. 4:131. 10.3389/fmicb.2013.00131 23734148 PMC3661993

[B38] Sadro S. Nelson C. E. Melack J. M. (2011). The influence of landscape position and catchment characteristics on aquatic biogeochemistry in high-elevation lake-chains. Ecosystems 15, 363–386 10.1007/s10021-011-9515-x

[B39] Santos A. L. Baptista I. Lopes S. Henriques I. Gomes N. C. M. Almeida A.. (2012a). The UV responses of bacterioneuston and bacterioplankton isolates depend on the physiological condition and involve a metabolic shift. FEMS Microbiol. Ecol. 80, 646–658. 10.1111/j.1574-6941.2012.01336.x 22356615

[B40] Santos A. L. Oliveira V. Baptista I. Henriques I. Gomes N. C. M. Almeida A.. (2012b). Effects of UV-B Radiation on the structural and physiological diversity of bacterioneuston and bacterioplankton. Appl. Environ. Microbiol. 78, 2066–2069. 10.1128/AEM.06344-11 22247171 PMC3298163

[B41] Sarmento H. Unrein F. Isumbisho M. Stenuite S. Gasol J. M. Descy J. P. (2008). Abundance and distribution of picoplankton in tropical, oligotrophic Lake Kivu, eastern Africa. Freshw. Biol. 53, 756–771 10.1111/j.1365-2427.2007.01939.x

[B42] Sarmento H. (2012). New paradigms in tropical limnology: the importance of the microbial food web. Hydrobiologia 686, 1–14 10.1007/s10750-012-1011-6

[B43] Schiaffino M. R. Gasol J. M. Izaguirre I. Unrein F. (2013). Picoplankton abundance and cytometric group diversity along a trophic and latitudinal lake gradient. Aquat. Microb. Ecol. 68, 231–250 10.3354/ame01612

[B44] Sherr B. F. del Giorgio P. Sherr E. B. (1999). Estimating abundance and single-cell characteristics of respiring bacteria via the redox dye CTC. Aquat. Microb. Ecol. 18, 117–131 10.3354/ame018117

[B45] Sieracki M. E. Cucci T. L. Nicinski J. (1999). Flow cytometric analysis of 5-cyano-2,3-ditolyl tetrazolium chloride activity of marine bacterioplankton in dilution cultures. Appl. Environ. Microbiol. 65, 2409–2417. 10347021 10.1128/aem.65.6.2409-2417.1999 PMC91356

[B46] Smith D. C. Azam F. (1992). A simple, economical method for measuring bacterial protein synthesis rates in seawater using 3H-leucine. Mar. Microb. Food Webs 6, 107–114.

[B47] Smith E. M. (1998). Coherence of microbial respiration rate and cell-specific bacterial activity in a coastal planktonic community. Aquat. Microb. Ecol. 16, 27–35 10.3354/ame016027

[B48] Sommaruga R. Obernosterer I. Herndl G. J. Psenner R. (1997). Inhibitory effect of solar radiation on thymidine and leucine incorporation by freshwater and marine bacterioplankton. Appl. Environ. Microbiol. 63, 4178–4184. 16535724 10.1128/aem.63.11.4178-4184.1997 PMC1389280

[B49] Sommaruga R. Psenner R. (1997). Ultraviolet radiation in a high mountain lake of the austrian alps: air and underwater measurements. Photochem. Photobiol. 65, 957–963 10.1111/j.1751-1097.1997.tb07954.x

[B50] Sommaruga R. Sattler B. Oberleiter A. Wille A. Wögrath-Sommaruga S. Psenner R. (1999). An *in situ* enclosure experiment to test the solar UVB impact on plankton in a high-altitude mountain lake. II. Effects on the microbial food web. J. Plankton Res. 21, 859–876 10.1093/plankt/21.5.859

[B51] Sommaruga R. (2001). The role of solar UV radiation in the ecology of alpine lakes. J. Photochem. Photobiol. B Biol. 62, 35–42. 10.1016/S1011-1344(01)00154-3 11693365

[B52] Suttle C. A. Chen F. (1992). Mechanisms and rates of decay of marine viruses in seawater. Appl. Environ. Microbiol. 58, 3721–3729. 16348812 10.1128/aem.58.11.3721-3729.1992 PMC183166

[B53] Triadó-Margarit X. Casamayor E. O. (2012). Genetic diversity of planktonic eukaryotes in high mountain lakes (Central Pyrenees, Spain). Environ. Microbiol. 14, 2445–2456. 10.1111/j.1462-2920.2012.02797.x 22672082

[B54] Vila-Costa M. Bartrons M. Catalan J. Casamayor E. O. (2014). Nitrogen-Cycling Genes in Epilithic biofilms of oligotrophic high-altitude lakes (Central Pyrenees, Spain). Microb. Ecol. 68, 60–69. 10.1007/s00248-014-0417-2 24743884

[B55] Zöllner E. Santer B. Boersma M. Hoppe H.-G. Jürgens K. (2003). Cascading predation effects of Daphnia and copepods on microbial food web components. Freshw. Biol. 48, 2174–2193. 10.1046/j.1365-2426.2003.01158.x24190166

