# Supplementary material for: Microbial food web components, bulk metabolism, and single-cell physiology of piconeuston in surface microlayers of high-altitude lakes
Source: Front Microbiol. 2015 May 5;6:361. doi: 10.3389/fmicb.2015.00361 (PMC4419848; doi:10.3389/fmicb.2015.00361)
Supplement: Supplementary file 1 [file Image1.PDF]

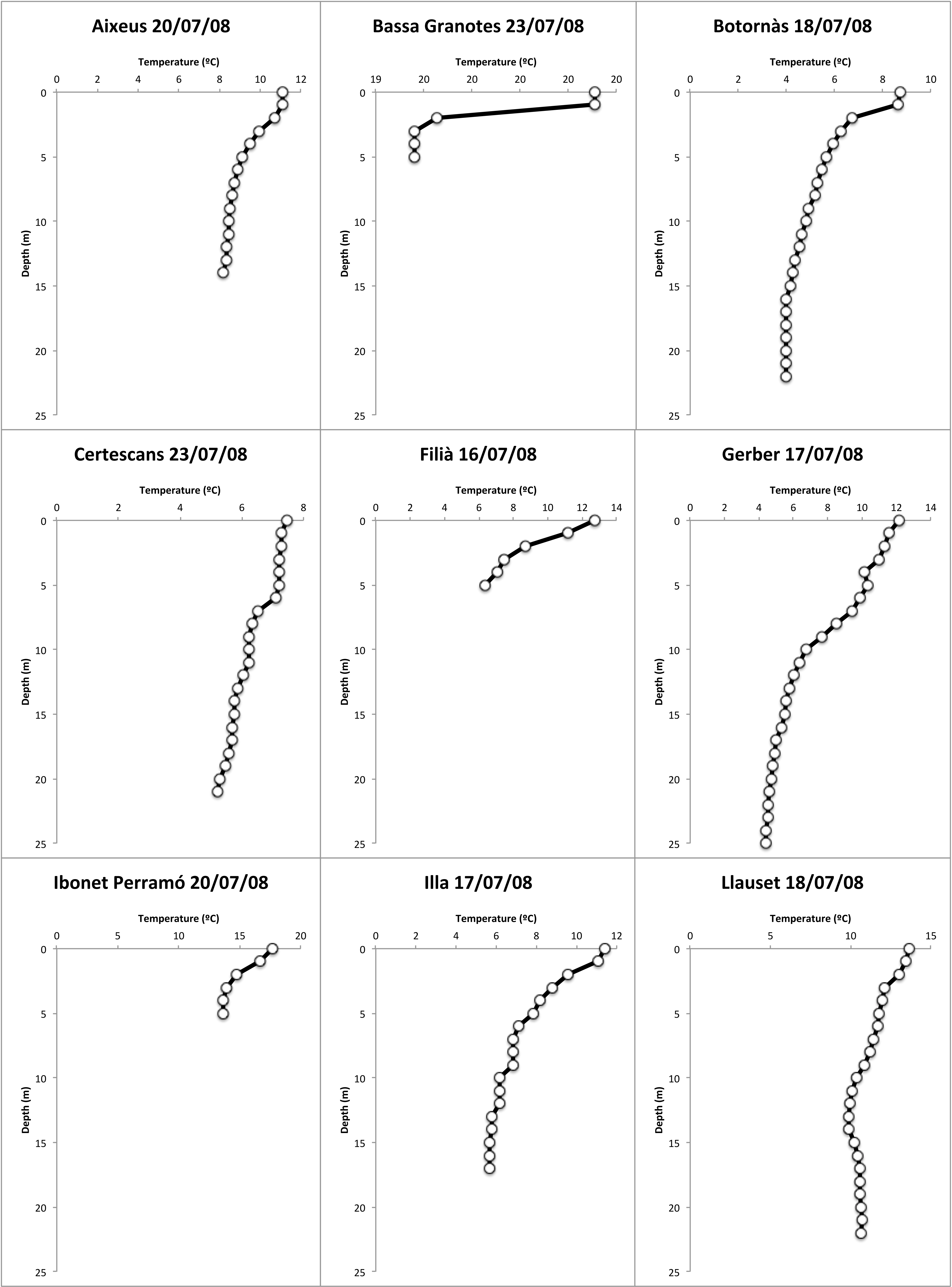

Aixeus 20/07/08
Temperature (ºC)
0 2 4 6 8 10 12
Depth (m)
0 5 10 15 20 25
Bassa Granotes 23/07/08
Temperature (ºC)
19 20 20 20 20 20
Depth (m)
0 5 10 15 20 25
Botornàs 18/07/08
Temperature (ºC)
0 2 4 6 8 10
Depth (m)
0 5 10 15 20 25
Certescans 23/07/08
Temperature (ºC)
0 2 4 6 8
Depth (m)
0 5 10 15 20 25
Filià 16/07/08
Temperature (ºC)
0 2 4 6 8 10 12 14
Depth (m)
0 5 10 15 20 25
Gerber 17/07/08
Temperature (ºC)
0 2 4 6 8 10 12 14
Depth (m)
0 5 10 15 20 25
Ibonet Perramó 20/07/08
Temperature (ºC)
0 5 10 15 20
Depth (m)
0 5 10 15 20 25
Illa 17/07/08
Temperature (ºC)
0 2 4 6 8 10 12
Depth (m)
0 5 10 15 20 25
Llauset 18/07/08
Temperature (ºC)
0 5 10 15
Depth (m)
0 5 10 15 20 25

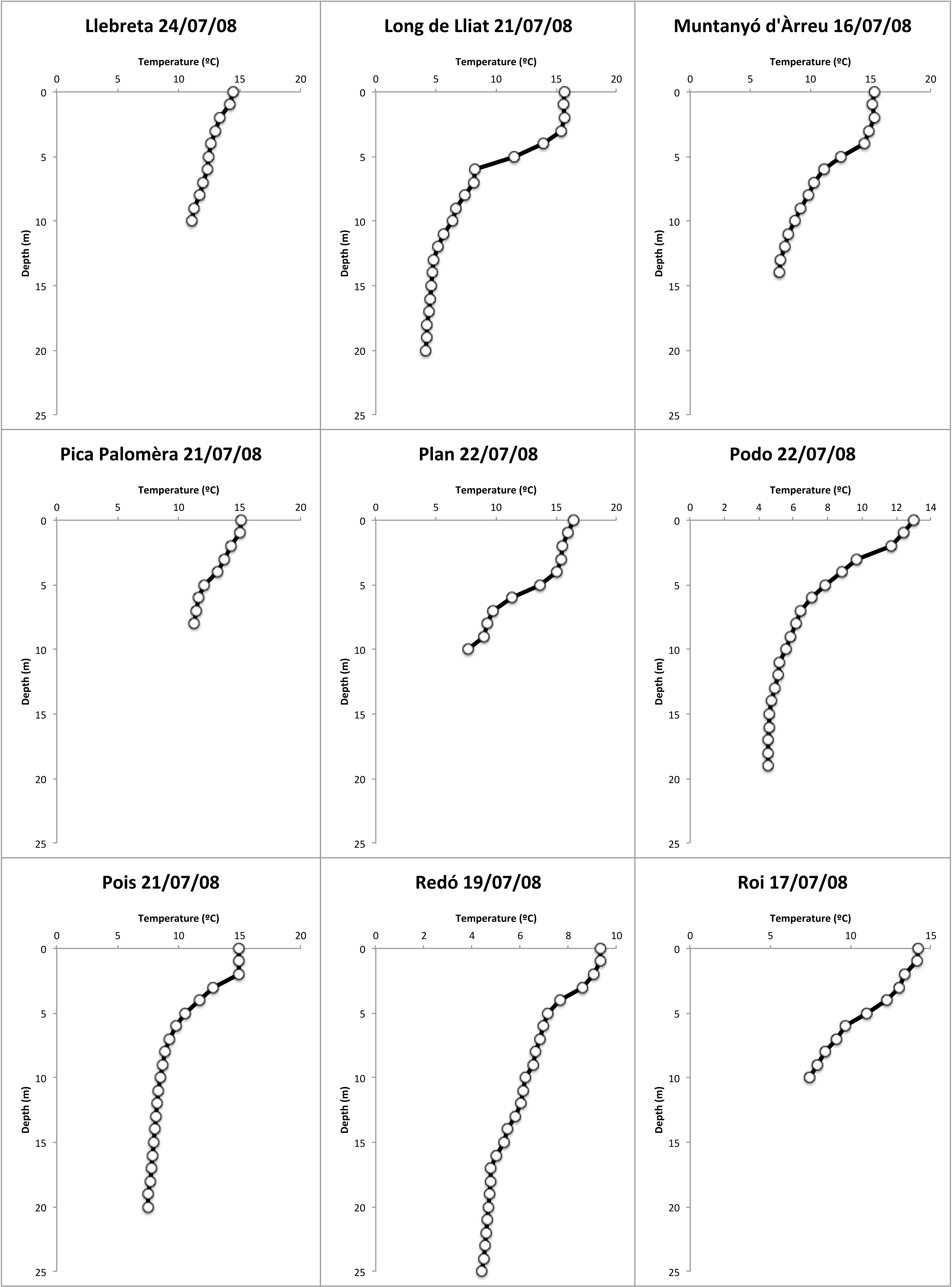

Llebreta 24/07/08
Long de Lliat 21/07/08
Muntanyó d'Àrreu 16/07/08
Pica Palomèra 21/07/08
Plan 22/07/08
Podo 22/07/08
Pois 21/07/08
Redó 19/07/08
Roi 17/07/08
Temperature (ºC)
Depth (m)

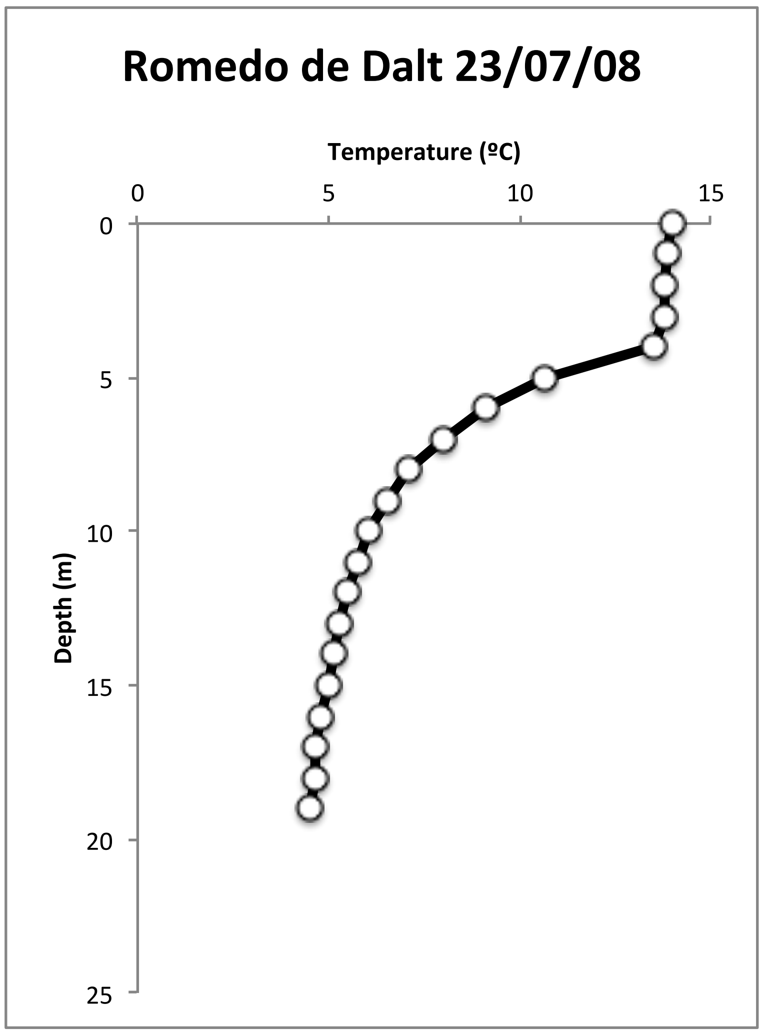
Romedo de Dalt 23/07/08
Temperature (ºC)
0
5
10
15
Depth (m)
0
5
10
15
20
25
